# 

**Published:** 1936

**Authors:** 


### The Medical Library of the University of Bristol,

WITH WHICH IS INCORPORATED

**The Library of the Bristol Medico-Chirurgical Society.**

*The following donations have been received since the publication of the list in June,* 1936.

October, 1936.

| | |
|---|---|
| American Otological Society (1) .. .. | 1 volume. |
| Professor R. J. Brocklehurst (2) .. .. | 3 volumes. |
| Michell Clarke Fund (3) .. .. .. .. | 23 volumes. |
| J. S. Cottrell & Co., Publishers (4) .. .. | 1 volume. |
| Henry Phipps Institute (5) .. .. .. | 1 volume. |

Unbound periodicals have also been received from Professor E. W. Hey Groves, Professor C. Bruce Perry and Dr. J. Odery Symes.

### THE ONE HUNDRED AND FIFTY-SIXTH LIST OF BOOKS.

The figures in round brackets refer to the figures after the names of the donors. The books to which no such figures are attached have either been bought from the Library fund or received through the *Journal.*

| | | | |
|---|---|---|---|
| **Auld, A. G.** | *Asthma, Hay Fever, Migraine* | | 1936 |
| **Bailey, Hamilton** | *Emergency Surgery* | 2nd Ed. | 1936 |
| **Bailey and Love** | *Short Practice of Surgery* | 3rd Ed. | 1936 |
| **Balme, H.** | *The Relief of Pain* | | 1936 |
| **Beaumont, G. E.** | *Medicine* | 2nd Ed. (3) | 1935 |
| **Beaumont, G. E., and Dodds, E. C.** | *Recent Advances in Medicine* | 8th Ed. (3) | 1936 |
| **Black, G. V.** | *Operative Dentistry.* Vol. I–III | 7th Ed. (3) | 1936 |
| **Borradaile, L. A.** | *A Manual of Elementary Zoology* | 8th Ed. | 1935 |
| **Browne, O'Donel** | *Manual of Practical Obstetrics* | | 1936 |
| **Buchanan, A. M.** | *Manual of Anatomy* | 5th Ed. (2) | 1925 |
| **Cameron, A. T., and Gilmour, C. R.** | *The Biochemistry of Medicine* | 2nd Ed. | 1935 |
| **Chamberlain, E. N.** | *Symptoms and Signs in Clinical Medicine* | | 1936 |
| **Cheesman, J. E.** | *Baillière's Synthetic Anatomy* | | 1936 |
| **Cope, Z.** | *Early Diagnosis of the Acute Abdomen* | 7th Ed. | 1935 |
| **Cruikshank, J. Norman.** | *Bright's Disease* | | 1933 |
| **Dilling, W. J.** | *Bruce and Dilling's Materia Medica and Therapeutics* | 14th Ed. (3) | 1935 |
| **Dingwall, E. J.** | *The Girdle of Chastity* | | 1931 |
| **East and Bain** | *Recent Advances in Cardiology* | 3rd Ed. (3) | 1936 |
| **Ennis, Le R. M.** | *Dental Roentgenology* | 2nd Ed. | 1936 |
| **Evans, C. Lovatt** | *Recent Advances in Physiology* | 5th Ed. | 1936 |
| **Gabell, D.** | *Prosthetic Dentistry* | 2nd Ed. (3) | 1936 |
| **Greenwood, M.** | *The Medical Dictator* | | 1936 |
| **Hadfield, C. J.** | *Practical Anæsthetics* | 2nd Ed. (3) | 1931 |
| **Haggard, H. W., and Greenberg, L. A.** | *Diet and Physical Efficiency* | | 1935 |
| **Hale-White, W.** | *Great Doctors of the Nineteenth Century* | | 1935 |
| **Halliburton, W. O., Hewitt, J. A., and Robson, W.** | *Essentials of Chemical Physiology.* 2 Copies | 12th Ed. (2) | 1929 |
| **Halliburton, W. O., and Macdowall, R. J. S.** | *Handbook of Physiology* | 34th Ed. | 1935 |
| **Hammond, T. E.** | *Vitality and Energy in Relation to the Constitution* | | 1936 |
| **Handley, W. S.** | *The Prevention of Cancer* | | 1936 |
| **Harrington, F. T.** | *Treatment of Asthma* | | 1936 |
| **Hartley, P. H.-S., and Aldridge, H. R.** | *Johannes de Mirfeld: His Life and Works* | | 1936 |
| **Holzer, W., and Weissenberg, E.** | *Foundations of Short-wave Therapy* | | 1935 |
| **Illingworth, C. F. W., and Dick, B. M.** | *A Text-Book of Surgical Pathology* | 2nd Ed. | 1935 |

**Joslin, E. P.** .. .. *Treatment of Diabetes Mellitus* 5th Ed. (3) 1935
**Kemble, J.** .. .. *Hero Dust* .. .. .. .. .. .. .. .. .. .. 1936
**King, E. S. J.** .. .. *Localized Rarefying Conditions of Bone* (3) 1935
**Lake, N. C.** .. .. *The Foot* .. .. .. .. .. .. .. .. .. (3) 1935
**Lewis, T.** .. .. .. *Vascular Disorders of the Limbs* .. .. .. 1936
**Martindale and Westcott.** *The Extra Pharmacopœia.* Vol. II 20th Ed. 1935
**May, C. H., and Worth, C.** *A Manual of Diseases of the Eye* 7th Ed. (3) 1934
**Meyer, W.** .. .. *Normal Histology and Histogenesis* .. .. 1935
**Mitchiner, P. H.** .. *The Modern Treatment of Burns and Scalds* (3) 1935
**Munch, J. C.** .. .. *Bioassays* .. .. .. .. .. .. .. .. .. .. 1931
**Pearson, W. J., and Wyllie, W. G.** *Recent Advances in Diseases of Children* .. .. .. .. .. .. 3rd Ed. (3) 1935
**Pugh, M. A.** .. .. *Squint Training* .. .. .. .. .. .. .. .. 1936
**Ray, M. B.** .. .. *Hydrotherapy and Climatotherapy* .. .. (3) 1936
**Roaf, H. E.** .. .. *A Text-Book of Physiology* .. .. 2nd Ed. 1936
**Rolleston, H.** .. .. *The Endocrine Organs in Health and Disease* .. 1936
**Samson, E.** .. .. *Progressive Practice* .. .. .. .. .. .. (4) 1936
**Schwartz, J. R.** .. .. *Cavity Preparation and Abutment Construction* .. .. .. .. .. .. .. .. .. (3) 1936
**Sheldon, J. H.** .. .. *Hœmochromatosis* .. .. .. .. .. .. (3) 1935
**Solis-Cohen, S., and Githens, T. S.** *Pharmacotherapeutics* .. 1928
**Starling, E. H.** .. *Principles of Human Physiology* 7th Ed. (3) 1936
**Stevenson, R. S.** .. *Recent Advances in Laryngology* .. .. (3) 1933
**Thomson-Walker, J.** *Surgical Diseases and Injuries of the Genito-Urinary Organs* .. .. .. 2nd Ed. (3) 1936
**Turner, A. L.** .. .. *Diseases of the Nose, Throat and Ear* 4th Ed. 1936
**Vaughan, J. M.** .. *The Anœmias* .. .. .. .. .. .. 2nd Ed. 1936
**Warwick and Tunstall.** *First Aid* .. .. .. .. .. .. .. 15th Ed. 1936
**Weiss, S., and others.** *Diseases of the Liver* .. .. .. .. (3) 1935
**Winton, T. R., and Bayliss, L. E.** *Human Physiology* .. 2nd Ed. 1936
**Woodwark, S.** .. .. *Manual of Medicine* .. .. 4th Ed. (3) 1935
**Yearsley, M.** .. .. *Le Roy est mort!* .. .. .. .. .. .. .. .. 1935

TRANSACTIONS, REPORTS, JOURNALS, ETC.

Journal of Obstetrics and Gynaecology of the British Empire. Index to Vols. XXXI–XI .. .. .. .. .. .. .. .. .. .. .. .. .. 1936
Henry Phipps Institute, 26th Report .. .. .. .. .. .. .. .. (5) 1935
Surgical Clinics of North America, June, August, 1936 .. .. .. 1936
Transactions of the American Otological Society. Vol. XXV (1) 1936
Transactions of the Medical Society, LIX .. .. .. .. .. .. .. 1936

### Publications Received

From Edward Arnold & Co. :

*Legal Problems in Medical Practice.* By Dr. Harcourt Kitchin of Gray's Inn, Barrister-at-Law. Price 10s. 6d.

*Manual of Pharmacology.* By W. E. Dixon. Price 18s.

From Baillière, Tindall and Cox :

*British Masters of Medicine.* Edited by Sir D'Arcy Power, K.B.E., F.R.C.S., F.S.A. Price 7s. 6d.

Cassell & Co. Ltd. :

*The Doctor.* By Mary Roberts Rinehart. Price 7s. 6d.

Eyre & Spottiswoode :

*Favourite Prescriptions.* Ed. Rolleston and Moncrieff. Price 10s. 6d.

From William Heinemann Ltd. :

*Research on the Low Potencies of Homœopathy* . . . . By W. E. Boyd, M.A., M.D. Price 2s. 6d.

*Maternity and Post-Operative Exercises in Diagrams and Words.* By Margaret Morris, C.S.M.M.G. Price 7s. 6d.

Hutchinson's Scientific and Technical Publications :

*Essentials of Modern Medical Treatment.* By Vincent Norman. Price 10s. 6d.

From H. K. Lewis & Co. Ltd. :

*Reports on Chronic Rheumatic Diseases* . . . . Edited by C. W. Buckley, M.D., F.R.C.P. No. 2. Price 10s. 6d.

*Diseases of the Testicle.* By Hamilton Bailey, F.R.C.S. Price 12s. 6d.

From Oxford University Press :

*An Introduction to Psychological Medicine.* By R. G. Gordon, M.D., D.Sc., F.R.C.P. (Ed.), N. G. Harris, M.D., B.S., D.P.M., and J. R. Rees, M.A., M.D., D.P.H. Price 10s. 6d.

*The Human Foot : Its Evolution, Physiology and Functional Disorders.* By Dudley J. Morton (Associate Professor of Anatomy, Columbia University). Price 15s.

*Home Care of the Mental Patient.* By Dr. Arie Querido. Price 2s. 6d.

*Health, Sickness and Psychology.* By Rev. R. W. Wilde. Price 3s. 6d.

*Paget's Disease of the Nipple.* By Keith Inglis. Price 36s.

From Williams & Norgate Ltd. :

*The Medical Dictator* . . . By Major Greenwood, F.R.S., D.Sc., F.R.C.P. Price 7s. 6d.

From John Wright & Sons :

*The British Journal of Surgery,* XXIV. No. 93. July, 1936. Price 12s. 6d.

### Local Medical Notes

APPOINTMENTS.

**University of Bristol.**—R. T. Cooke, M.D., Assistant Clinical Pathologist, Department of Preventive Medicine ; C. A. H. Gee, M.B., Ch.B., Clinical Lecturer in Diseases of Children ; L. C. Oliver, F.R.C.S., Recognized Teacher in Surgery ; P. Phillips, M.D., Recognized Teacher in Obstetrics ; C. A. Ashford, M.A., Ph.D., Lecturer in Physiology ; Miss J. Wiseman, B.Sc., Assistant Bacteriologist, Department of Preventive Medicine ; H. Tout, M.A., Colston Research Fellow in Social Survey.

**Bristol Royal Infirmary.**—W. Melville Capper, M.R.C.S., L.R.C.P., M.B., B.S., F.R.C.S., Senior Resident Medical Officer.

**Bristol General Hospital.**—L. C. Oliver, F.R.C.S., Resident Surgical Officer and Registrar.

EXAMINATION RESULTS.

**University of Bristol.**—Students of the University have recently passed the following examinations :—

M.B., Ch.B.—*First Examination. Pass :* J. T. N. Cole, D. S. Maunsell, Phyllis D. Moses.

B.D.S.—*First Examination. Pass :* F. C. Baker, K. L. Brown.

L.D.S.—*Preliminary Science Examination. Pass :* H. C. Davey, L. Murray, J. R. Herbert.

**University of Cambridge.**—M.B., B.S. (*Part I.*). *Pass :* C. J. F. Coombs.

**Conjoint Board.**—L.R.C.P., M.R.C.S. *Pass : In Anatomy and Physiology :* Mary R. More, E. W. J. Townsend. *In Anatomy :* C. J. R. Jacob, Joyce S. Miller, J. H. G. Morris. *In Physiology :* J. I. Williams. *In Pathology and Medicine :* J. A. Cochrane.* *In Medicine :* W. H. Hayes.* *In Midwifery :*

* Qualified.

Margaret A. W. Hawkes, F. D. Mowat. *In Pathology :* J. H. Bulleid, E. J. Lace, Mabel W. N. Tribe.

L.D.S., R.C.S.—*Final Examination.* (*Part II.*) *Pass :* A. N. Brimelow,* J. T. S. Hutchins,* C. C. B. Plumley,* W. White.*

* Qualified.

---

**The Long Fox Memorial Lecture.**—The twenty-fifth Long Fox Memorial Lecture was delivered at 8.30 p.m. on Tuesday, 20th October, in the University, by Dr. A. Bulleid, F.S.A. Subject : "Somerset Lake Villages."

